# Serum Level of D-Lactate in Patients with Cystic Fibrosis: Preliminary Data

**DOI:** 10.1155/2018/5940893

**Published:** 2018-07-12

**Authors:** Sabina Więcek, Jerzy Chudek, Halina Woś, Maria Bożentowicz-Wikarek, Bożena Kordys-Darmolinska, Urszula Grzybowska-Chlebowczyk

**Affiliations:** ^1^Department of Pediatrics, School of Medicine in Katowice, Medical University of Silesia, Katowice, Poland; ^2^Pathophysiology Unit, Department of Pathophysiology, School of Medicine in Katowice, Medical University of Silesia, Katowice, Poland; ^3^Department of Internal Medicine and Oncological Chemotherapy, School of Medicine in Katowice, Medical University of Silesia, Katowice, Poland; ^4^University of Bielsko-Biała, Bielsko-Biała, Poland

## Abstract

D-Lactate is produced by the intestinal biota and later absorbed into circulation. Some patients with cystic fibrosis (CF) develop exocrine pancreatic insufficiency that may disturb the gut microbiome and enhance the production of D-lactate. However, this concept has not been studied yet. The aim of the study was to assess D-lactate concentration in relation to the occurrence of clinical features, activity of CF, and diet composition in paediatric patients. *Patients and Method*. Serum concentrations of D-lactate were measured in 38 CF patients (19 girls and 19 boys) from 6 months to 18 years of age. The analysis included age, sex, clinical symptoms, diet (the variety and calorie needs), the laboratory tests for pancreatic efficiency (serum levels of albumin and glucose, faecal elastase activity, and faecal fat index) and faecal calprotectin (the marker of intestinal inflammation), and parameters of liver damage and of cholestasis (the activity of aminotransferases, *γ*-glutamyltransferase, level of bilirubin, and international normalized ratio). *Results*. The median level of D-lactate was 0.86 *μ*g/ml (1Q–3Q: 0.48–2.03) and correlated with the CF severity in the Schwachman-Kulczycki score, parameters of pancreatic insufficiency, and the presence of intestinal inflammation. An increased level of D-lactate was observed in the subgroup with pancreas insufficiency (1.05 versus 0.73; *p* < 0.05), parallel with an elevated level of calprotectin (0.948 versus 0.755; *p* = 0.08). There was no relationship between energy consumption and diet composition and serum D-lactates. *Conclusion*. Serum D-lactate concentration in CF patients is a promising new marker of exocrine pancreatic insufficiency probably related to intestinal flora dysbiosis/overgrowth.

## 1. Introduction

Cystic fibrosis (CF) is the most common genetic, multiorgan chronic condition in Caucasians. It originates from inherited mutations of the cystic fibrosis transmembrane conductance regulator *(CFTR)* gene, which encodes a transmembrane protein with a functional chloride channel. A dysregulation of the chloride channel activity results in systemic dysfunction of exocrine secretion glands manifested by a variety of gastrointestinal and respiratory symptoms. The exocrine pancreatic insufficiency results from the abnormal secretion of water, bicarbonates, and chloride ions that develops in 80–90% of patients. Chronic fatty diarrhoea is the most frequent clinical symptom. Exocrine pancreatic insufficiency, disorders of motility of the gastrointestinal tract, and the lack of appetite very often precipitate the development of malnutrition. As a result of a large volume of partially digested nutrients, the presence of thick glycoprotein-rich mucus changes the components of the intestinal flora, and due to abnormalities of gut motility, small intestinal bacterial overgrowth (SIBO) often develops. Prolonged antibiotic therapy given to patients due to frequent bronchopulmonary complications is an additional factor in the development of the condition [1–4]. Intestinal dysbiosis and bacterial overgrowth occur in 30–50% of CF patients [5, 6]. However, SIBO may not be recognised due to similar clinical manifestations of CF disorders and can promote development of malnutrition.

As mentioned above, exocrine pancreatic insufficiency predisposes to bacterial overgrowth, dysbiosis, and fermentation of the abnormally digested food. *Escherichia coli*, *Enterococcus* spp., and *Klebsiella pneumoniae* are bacterial strains dominant in SIBO that suppress the growth of *Lactobacillus* and *Bifidobacterium* [7–10]. Gut microbiota (*Lactobacillus*, *Escherichia coli*, *Klebsiella*, and *Bacteroides*) are the main producers of D-lactate in the human body, which is later absorbed into the bloodstream and excreted into urine, as D-lactate unlike L-lactate is not metabolised by the human body due to a lack of relevant enzymes [11–15]. Therefore, it seems that changes in the composition of intestinal flora (dysbacteriosis) may be reflected by elevated serum levels of D-lactate in CF patients, especially those with exocrine pancreatic insufficiency. Serum levels of D-lactate have not been assessed in patients with CF, yet.

The aim of the study was to assess D-lactate concentration in relation to the occurrence of clinical features, activity of CF, and diet composition in paediatric patients.

## 2. Patients and Method

The study included a group of 38 children (19 girls and 19 boys) 6 months to 18 years of age (the average age of 7.8 years), admitted to the Department of Paediatrics or monitored in the Cystic Fibrosis Outpatient Clinic. The inclusion criteria involved the diagnosis of CF confirmed by genetic testing. Each patient or his/her legal guardian signed informed consent to participate in the study. Patients aged less than 6 months as well as those with acute infection or who received antibiotics for any cause during the previous 4 weeks were excluded.

The study protocol was approved by the Bioethics Committee of the Silesian Medical University in Katowice.

The following investigations were performed during hospitalisation: nutrition status (body mass, height, and BMI), pancreatic efficiency (serum levels of albumin and glucose, faecal elastase activity, and faecal fat index), and faecal calprotectin level (normal range < 50 *μ*g/1 g stool), as well as liver and cholestasis parameters (the activity of aminotransferases, GGTP, and coagulation parameters). All patients also underwent an abdominal ultrasound.

Following the receipt of the patient's consent, a blood sample was obtained for routine tests, after overnight fast (at least 8 hours), and excess serum was frozen in order to assess the concentration of D-lactate.

In addition, the composition of the diet was analysed on the basis of a 3-day diary. Caloric protein, carbohydrate, and fat intakes were converted to per-kilogram body weight of the patient.

Concentration of D-lactate was determined using the ELISA method (USCN Life Sciences Inc., Wuhan, China) with the intra-assay coefficients of variability < 8%.

The severity of CF was assessed based on the Schwachman-Kulczycki score including information on physical activity and abnormalities in the respiratory tract, nutrition, and imaging tests [2, 3].

Exocrine pancreatic insufficiency was diagnosed based on clinical symptoms and laboratory tests (faecal fat index > 10% and faecal elastase activity < 200 *μ*g/g). The patients' nutrition states were determined using growth charts for body weight, height, and BMI specific for the Polish population. BMI below 3% for age and sex was considered to be malnutrition. For children aged less than 2 years, we used special growth charts for small children.

### 2.1. Statistical Analysis

The analysis was compliant with the procedures set forth in the MedCalc 14.8.1 licenced software (MedCalc Software, Ostend, Belgium). Quantity variants of normal distribution were presented as medians with interquartile range, due to nonparametric distribution of D-lactate concentrations. Quality variables were presented as absolute value and the proportion.

The inter-subgroup (distinguished by age, sex, clinical symptoms, results of laboratory tests assessing exocrine and endocrine pancreatic efficiency, parameters of liver damage, and cholestasis and dietary habits) differences in the concentration of D-lactate were assessed with the Mann–Whitney *U* test. The chi-square test or Fisher's exact test was used for quality variants. Correlation coefficients were calculated according to Spearman. The statistical significance was established at *p* < 0.05.

## 3. Results

The most commonly observed mutations of *CFTR* were delF508, with homozygous delF508/delF508 in 22 patients (57.9%), and heterozygous delF508/other in 13 patients (34.2%).

The clinical presentation of CF patients was shown in Table 1. The symptoms of exocrine pancreatic insufficiency occurred in 29 children (76.3%), recurrent infections of the respiratory tract in 23 patients (60.5%), and malnutrition in 18 (47.4%). The average severity of CF on the Schwachman-Kulczycki score was 74.2.

### 3.1. Serum D-Lactate and Clinical Course of CF

The median of serum D-lactate was 0.86 *μ*g/ml (1Q–3Q: 0.48–2.03) and was not age-dependent.

There was no correlation between serum concentration of D-lactate and diet composition, which included the intake of calories from protein, carbohydrate, and fat.

The highest serum levels of D-lactate were observed in patients with pancreatic insufficiency (Table 2). Serum concentrations of D-lactate correlated with the Schwachman-Kulczycki score values (*R* = −0.393; *p* = 0.01). As far as the CFTR gene mutation is concerned, the highest concentration of D-lactate was found in patients with the homozygous mutation delF508/delF508 which correlated with the most severe course of the disease: 0.95 (1Q–3Q: 0.67–1.03). The values were lower in the heterozygote cases: 0.76 *μ*g/ml (1Q–3Q 0.64–0.93) (Table 2).

**Figure 1:**
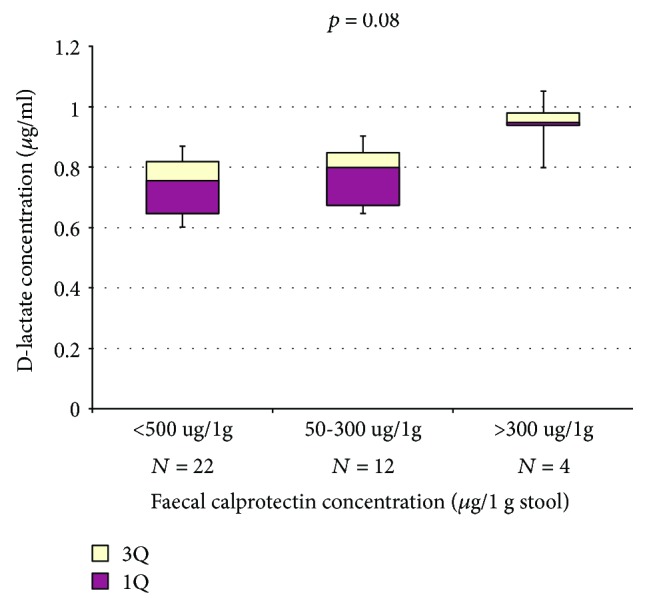
Serum levels of D-lactate in relation to the concentration of faecal calprotectin (normal range < 50 *μ*g/1 g of stool).

Increased concentration of faecal calprotectin (>50 *μ*g/1 g stool) was found in 16 patients (42.1%)—all with diagnosed pancreatic insufficiency. However, in relation to those with normal faecal calprotectin levels, the difference in D-lactate did not reach statistical significance (0.948 versus 0.755 *μ*g/ml; *p* = 0.08) (Figure 1). The correlation between serum D-lactates and faecal calprotectin was not statistically significant (*R* = 0.29; *p* = 0.09).

### 3.2. Serum D-Lactate and Diet

There was no relationship between energy consumption and diet composition and serum D-lactates (data not shown).

## 4. Overview and Discussion

To the best of our knowledge, this is the first description of serum D-lactate levels in patients with CF. We found that the highest levels of D-lactate are observed in patients suffering from exocrine pancreatic insufficiency, while other clinical symptoms are not affecting them.

There are known clinical conditions that may affect serum levels of D-lactate, for example, prolonged antibiotic therapy of recurrent respiratory infections, and that may affect the growth of *Lactobacillus* which produces D-lactate [16, 17]. Therefore, we put much effort to eliminate the influence of potential confounders, and we excluded all patients treated with antibiotics during the previous 4 weeks and assessed the serum concentration of D-lactate in the early morning after an overnight fast.

We did not analyse faecal microbiota; however, we measured faecal calprotectin concentrations that are related to small intestine bacterial overgrowth in CF patients, which was found to decrease after the use of probiotics (*Lactobacillus casei* strain GG). In our study, we showed that elevated serum levels of D-lactate are proportional to the concentrations of faecal calprotectin in CF patients. Probably due to the size of our study group, the association was of borderline significance.

Furthermore, serum D-lactate concentrations correlated with CF activity (reflected by Schwachman-Kulczycki score values). The highest concentrations of D-lactate were noticed in patients with homozygous delF508/delF508 mutations, which characterised those with the most severe clinical conditions. The available literature was focused on the increase in the serum levels of D-lactate in patients with the malabsorption syndrome and exocrine pancreatic insufficiency in non-CF patients, which supports our results [15, 18]. Outside of CF, elevated serum levels of D-lactate were observed in patients with severe gastrointestinal tract infections [11] and in those with short bowel syndrome, despite the lack of acidosis, and were likely to be related to absorption and digestion abnormalities caused by the underlying condition [16, 19, 20]. Serum D-lactate may be additionally affected by coexisting lactose intolerance and bacterial overgrowth in the small intestine [19, 21, 22].

Contrary to exocrine pancreatic insufficiency, there was no association between serum D-lactates and the occurrence of malnutrition that was highly prevalent—almost in every second study subject. Malnutrition in CF patients is the result of numerous factors, including the already mentioned exocrine pancreatic insufficiency and the lack of appetite, frequent in chronic conditions. This may decrease consumption of recommended diet, high in calories (120–150% higher than that of a healthy peer) and composed of protein, fat, and carbohydrates.

A link between excessive intakes of carbohydrates, which ferment in the intestines and elevate concentration levels of serum D-lactate, is well known in non-CF patients [23–26]. Nevertheless, it was not observed among our patients; that might be caused by the fact that our study cohort has been under constant dietary supervision (reflected by well-balanced diet composition).

The observed values of D-lactate in CF subjects with exocrine pancreatic insufficiency may be considered as a potential new marker of exocrine pancreatic insufficiency but without any influence on the clinical course of the disease. The levels of serum D-lactate were too low to trigger neurological symptoms. Headaches, tiredness, anxiety and behavioural changes, ataxia, slower speech, impaired consciousness, and blurred vision resulting from encephalopathy were reported in patients with short intestine syndrome and following jejuno-ileal bypass with serum levels of D-lactate exceeding 2.5–3.0 mmol/l (222–267 *μ*g/ml) [27].

Our study has some limitations related to the size of the CF cohort and because the hydrogen breath test to diagnose SIBO was performed only in 5 patients. Low number of some subgroups precluded achievement of statistical significance. Therefore, our study should be considered as preliminary, at least in some aspects.

In conclusion, serum D-lactate concentration in patients with cystic fibrosis is a promising new marker of exocrine pancreatic insufficiency probably related to intestinal flora dysbiosis/overgrowth.

## Figures and Tables

**Table 1:** Clinical characteristics and diet composition of 43 patients with cystic fibrosis. Diet composition was expressed as median and 1–3 quartiles.

	Values
*Age (years)*	7.8 (range: 6 months–18 years)
*Sex: female/male (n/n)*	19/19
*Mutation (n (%))*	
delF508del/delF508del	22 (57.8)
delF508del/other	13 (34.0)
Other	3 (7.9)
*Schwachman-Kulczycki score (pts)*	74.2 (range 45–110)
*Clinical features (n (%))*	
Pancreatic insufficiency	29 (76.3)
Malnutrition (BMI < 3 pcn)	18 (47.4)
Symptoms from respiratory tract/recurrent respiratory tract infection	23 (60.5)
Liver dysfunction	7 (18.4)
History of meconium ileus/treated surgically	3 (7.9)/1 (2.6)
Salt-loss syndrome	2 (5.2)
Elevated concentration of faecal calprotectin	16 (42.1)
*Diet composition*	
Energy (kcal/kg body mass)	70.7 (55.8–90.4)
Protein (g/kg body mass)	2.4 (1.8–3.3)
Carbohydrates (g/kg body mass)	8.5 (6.8–10.6)
Fat (g/kg body mass)	1.9 (1.6–3.7)
*Dietary support*	
PEG	2 (5.2%)
Nasogastric tube	3 (7.8%)
Orally high caloric formula	25 (65.78%)

**Table 2:** Serum levels of D-lactate in relation to clinical symptoms of cystic fibrosis (median: 1–3 quartiles).

		Serum D-lactate (*μ*g/ml)	Statistical significance
*CFTR* mutation			
delF508del/delF508del	*N* = 22	0.95 (0.67–1.03)	*p* = 0.09
delF508del/other	*N* = 13	0.76 (0.64–0.93)	
Other	*N* = 3	0.69 (0.60–0.78)	

Pancreatic insufficiency	Yes *N* = 29	1.05 (0.74–1.20)	*p* < 0.05
	No *N* = 9	0.73 (0.63–0.87)	

Malnutrition	Yes *N* = 18	0.85 (0.64–1.06)	*p* = 0.17
	No *N* = 20	0.77 (0.71–0.88)	

Liver dysfunction	Yes *N* = 7	0.70 (0.65–0.97)	*p* = 0.75
	No *N* = 31	0.76 (0.65–0.95)	

Symptoms from respiratory tract/recurrent respiratory tract infection	Yes *N* = 23	0.77 (0.67–1.02)	*p* = 0.40
	No *N* = 15	0.75 (0.63–0.94)	

Salt-loss syndrome	Yes *N* = 2	0.95 (0.72–1.22)	*p* = 0.08
	No *N* = 36	0.73 (0.64–0.94)	
